# Overexpression of a Novel Apple NAC Transcription Factor Gene, *MdNAC1*, Confers the Dwarf Phenotype in Transgenic Apple (*Malus domestica*)

**DOI:** 10.3390/genes9050229

**Published:** 2018-04-27

**Authors:** Dongfeng Jia, Xiaoqing Gong, Mingjun Li, Chao Li, Tingting Sun, Fengwang Ma

**Affiliations:** State Key Laboratory of Crop Stress Biology for Arid Areas/Shaanxi Key Laboratory of Apple, College of Horticulture, Northwest A&F University, Yangling, Shaanxi 712100, China; dongfengjia@163.com (D.J.); gongxq0103@nwsuaf.edu.cn (X.G.); limingjun@nwsuaf.edu.cn (M.L.); cl8609@nwafu.edu.cn (C.L.); suntingitng@126.com (T.S.)

**Keywords:** apple, *MdNAC1*, dwarf, abscisic acid, brassinosteroid

## Abstract

Plant height is an important trait for fruit trees. The dwarf characteristic is commonly associated with highly efficient fruit production, a major objective when breeding for apple (*Malus domestica*). We studied the function of *MdNAC1*, a novel NAC transcription factor (TF) gene in apple related to plant dwarfing. Localized primarily to the nucleus, MdNAC1 has transcriptional activity in yeast cells. Overexpression of the gene results in a dwarf phenotype in transgenic apple plants. Their reduction in size is manifested by shorter, thinner stems and roots, and a smaller leaf area. The transgenics also have shorter internodes and fewer cells in the stems. Levels of endogenous abscisic acid (ABA) and brassinosteroid (BR) are lower in the transgenic plants, and expression is decreased for genes involved in the biosynthesis of those phytohormones. All of these findings demonstrate that *MdNAC1* has a role in plants dwarfism, probably by regulating ABA and BR production.

## 1. Introduction

Apple (*Malus domestica*) is one of the most widely cultivated fruit trees worldwide. In modern orchards, the use of dwarf trees is an effective way to meet greater consumer demands. Although methods of traditional crosses or genetic modification are often applied to create dwarf varieties, the dwarfing mechanism has not been completely elucidated [1].

Plant dwarfism is a complex trait controlled by multiple genes [2]. *Arabidopsis thaliana* (hereafter *Arabidopsis*) mutant *sax1* shows a dwarf phenotype, delayed development, and reduced fertility [3]. Overexpression of the *Arabidopsis* gene *gai* in apple significantly decreases the overall size of plants because they have shorter stems and internodes and fewer nodes [4]. The loss-of-function mutations of *ACL5* lead to severe dwarfing and less extensive stem elongation in *Arabidopsis* [5]. Two quantitative trait loci (QTLs), *Dw1* and *Dw2*, are primarily responsible for rootstock-induced dwarfing in apple [2].

This phenotype is usually a result of reduced cell division and/or cell elongation. These processes are generally regulated by plant hormones, including cytokinins (CTKs) such as benzyladenine (BA), auxins such as indole acetic acid (IAA) and indole-3-butyric acid (IBA), gibberellin (GA), abscisic acid (ABA), and brassinosteroid (BR) [6,7,8]. An optimal level of ABA is required for normal plant growth, and a deficiency results in poor development [9,10]. For example, the ABA-deficient mutant *notabilis* in tomato (*Lycopersicon esculenium*) shows markedly impaired shoot growth and reduced root development [8]. Another tomato ABA-deficient mutant, *sitiens*, also displays dwarfism, with decreased leaf area and a lower relative growth rate [9]. Mutations in the BR pathway also cause distorted growth. For example, *dwarf 7-1* invalidates BR biosynthesis and mutant *Arabidopsis* plants exhibit slower rates of cell division and shoot induction [11]. In transgenic apple, overexpression of *MdWRKY9* significantly suppresses the expression of *MdDWF4*, which then decreases the level of BR and leads to dwarfing in apple rootstock M26 (*Malus pumila*) [7]. Furthermore, an *Arabidopsis* mutant, *dwf4*, shows a completely dwarfed phenotype, but application of BR rescues that to a wild-type (WT) phenotype, which suggests that the dwarfing is dependent upon the BR pathway [12].

Plant transcription factors (TFs) are important regulators that activate or inhibit transcription of downstream genes during plant development and in response to environmental stimuli. Multiple TFs can also have a role in dwarfism, including WRKY, AP2, BHLH, ARF, and NAC [13,14,15]. The plant NAC proteins are essential for numerous biological processes, e.g., development, senescence, morphogenesis, and stress signal transduction [16]. Several NAC TFs induce dwarfism or growth inhibition. In *Arabidopsis*, overexpression of *NAC* genes *XND1*, *VND7*, and *ANAC036* cause a dwarf or semi-dwarf phenotype [15,17,18]. The same is true when *OsNAC6* from rice (*Oryza sativa*) is over-expressed, and overexpression of another NAC TF gene, *OsSWN1*, also causes a semi-dwarf phenotype in transgenic rice plants [19,20]. In poplar (*Populus trichocarpa*), overexpression of *PopNAC122* reduces overall plant height growth as well as the size and number of cells [21].

However, despite these great discoveries, the mechanisms underlying plant dwarfism are still poorly understood. In this study, we cloned a novel gene in apple according to a BLAST search result from Genome Database for Rosaceae (GDR) [22] and identified MdNAC1 as similar to NAC proteins from some other species. Its overexpression leads to a dwarf phenotype in apple and affected plants have significantly shorter shoots and roots, and smaller leaf areas when compared with the wild-type (WT). Our analytical results also suggested that *MdNAC1* confers this phenotype by regulating the biosynthesis of ABA and BR. These findings provide new insight into dwarfism and reveal *MdNAC1* as a valuable genetics resource for modern apple production.

## 2. Materials and Methods

### 2.1. Plant Materials and Growth

For expression assays in dwarfing and nondwarfing trees, three dwarfing rootstocks (M9, SH2, and T337) and three nondwarfing rootstocks (*Malus zumi*, *Malus pruifolia*, and *Malus robusta*) were used. Leaves were selected from these 10-year-old trees in mid-June and were immediately frozen in liquid nitrogen and stored at −80 °C until use.

We used GL-3 apple (*Malus domestica*) for gene transformation because it is more easily regenerated and is sensitive to *Agrobacterium tumefaciens* [21]. Plants were cultured in vitro on an MS medium (Murashige and Skoog medium) containing 0.3 mg L^−1^ 6-BA and 0.2 mg L^−1^ IAA at 23°C/20°C (day/night) and under a 16 h photoperiod (light intensity of 100 μmol m^−2^s^−1^). The plants were sub-cultured every 30 d. For rooting, GL-3 WT and transgenic plants were first transferred to an MS medium containing 0.5 mg L IBA^−1^ and 0.5 mg L^−1^ IAA and held under darkness for 15 d. After they were transferred to fresh MS media and rooted for one month, they were transplanted to organic substrate in pots and watered with ^1^/_2_-strength Hoagland nutrient solution every 4 d. The incubator conditions included 24°C/20°C (day/night), 16 h photoperiod, and a light intensity of 100 μmol m^−2^s^−1^. After another month, plants of uniform size from each genotype were moved to plastic pots containing a mixture of forest soil and organic substrate (5:1, v:v), and were exposed to natural, outdoor conditions for another month. Finally, they were transferred to the greenhouse where the experiments were conducted. During the treatment period, all of the plants were watered thoroughly to maintain a field capacity of 75%–85%. A total of 40 plants for each line were used in the experiments. The experiments were conducted for 90 d, between mid-July and mid-October, in a greenhouse at Northwest A & F University, Yangling (34°20′ N, 108°24′ E), China.

### 2.2. Isolation of MdNAC1 from Apple

Total RNA was isolated using leaves of *M. domestica* “Golden Delicious”. First-strand cDNA was synthesized using a RevertAid First Strand cDNA synthesis Kit (Fermentas, Theromo Scientific, Waltham, MA, USA). The target gene was amplified by polymerase chain reactions (PCRs), using primers *MdNAC1*cF and *MdNAC1*cR (Table S1), designed according to the predicted sequence from GDR. After ligation into pMD19-T simple vector, the product was validated by sequencing. The protein was then verified using the online hidden Markov model (HMM) [23], and the sequence was submitted to the GenBank database [24] and obtained an accession number of MF401514.1.

### 2.3. Phylogenetic Analysis, Multiple Alignments, and Examination of Gene Structure

Using NCBI [25], we downloaded 18 NAC protein sequences from different species that are homologous to MdNAC1 (Table S2). The phylogenetic analysis was performed with MEGA 5.50 software, using the neighbor-joining method with 1000 bootstrap replicates [26]. All protein sequences were aligned with DNAMAN software (version 6.0; Lynnon Biosoft, San Ramon, CA, USA). The gene structure of *MdNAC1* was analyzed based on the presence of exons and introns.

### 2.4. Subcellular Localization and Analysis of Transcriptional Activity for MdNAC1

The coding sequence of *MdNAC1*, without stop codon, was amplified with *att*B-containing primers *MdNAC1*slF and *MdNAC1*slR (Table S1), and a BP recombination reaction between the PCR products and the *att*P-containing donor vector pDONR^TM^222 was performed to generate an entry clone. Afterward, an LR recombination reaction was conducted with the entry clone and the *att*R-containing pGWB405 vector to generate a recombined vector with a C-terminus green fluorescent protein (GFP) tag. The 35S::MdNAC1‒GFP destination plasmid was introduced into *A. tumefaciens* strain EHA105, and leaves of tobacco (*Nicotiana benthaamiana*) were transiently transformed as described by Yang et al. [27]. After 2 d, GFP expression was observed using confocal microscopy and images were processed with FV10-ASW software (version 2.1a; Olympus, Berlin, Germany).

We also amplified the coding region of *MdNAC1* with primers *MdNAC1*tfF and *MdNAC1*tfR (Table S1) and inserted the product into the pGBKT7 vector between the *Eco*RI and *Bam*HI sites to generate a pGBKT7‒MdNAC1 recombined vector. Both pGBKT7‒MdNAC1 and pGBKT7 (negative control) were transformed by the method of Fujita et al. [28] into the yeast strain AH109, which harbored the *LacZ* and *HIS3* reporter genes. The transformed yeast cultures were dropped onto SD-Trp medium, and SD-Trp-His-Ade mediums (Synthetic Defined medium) with or without X-α-Gal and incubated for 3 d at 30 °C.

### 2.5. Generation of Transgenic Apple Plants

The coding sequence of *MdNAC1* was amplified with *att*B-containing primers *MdNAC1*F and *MdNAC1*R (Table S1), and the PCR products were first cloned into donor vector pDONR^TM^222 through a BP recombination reaction to generate an entry clone. Afterward, *MdNAC1* was transferred from the entry clone into the expression vector pGWB411, a plant expression vector driven by 35S promoter, via an LR recombination reaction. The recombinant pGWB411‒*MdNAC1* was transformed into *A. tumefaciens* strain EHA105 and GL-3 plants were used in vitro to generate transgenic apple plants. *Agrobacterium*-mediated transformation was performed according to the method described by Dai et al. [29]. Transgenic plants were confirmed by PCR analysis using vector-specific primers *att*B1 and *att*B2, and a pair of one gene-specific primer (*MdANC1*S) and one vector-specific primer (*att*B2) (Table S1).

### 2.6. qRT-PCR Analysis and Promoter Element Analysis

Expression of *MdNAC1* and genes involved in the biosynthesis and signaling pathways of ABA and BR was detected by quantitative real-time PCR (qRT-PCR) in transgenic and WT GL-3 plants. We referred previous reports to analyze the gene expressions of apple, *MdDWF4*, *MdCPD*, *MdBRox-1*, and *MdBRox-2*. In addition, some protein sequences of *Arabidopsis* genes were used to identify their homologous genes in apple, i.e., *MdNCED3*, *MdABI1-1*, *MdABI1-2, MdMYB2*, *MdMYC2*, and *MdRD22*. The detailed information was shown in Table S4. Total RNA was isolated from leaf samples and treated with RNase-free DNase I (Invitrogen, Carlsbad, CA, USA) to remove the DNA. Reverse-transcription was performed with a PrimeScript^®^ Reverse Transcriptase Kit (Takara, Kyoto, Japan). This qRT-PCR analysis was performed with SYBR^®^ Premix Ex Taq (Takara) using an ABI 7500 real-time PCR machine (Applied Biosystems, Carlsbad, CA, USA). All primers were designed according to the coding sequences of the different genes (Table S3), and *MdActin* (EB136338.1) served as an internal control. Each experiment was independently repeated four times. The delta–delta Ct method was used for qRT-PCR analysis. The specificities of all the primers were confirmed by PCR with correct predicted length and further by sequencing, and their corresponding melting curves with a single sharp. The primers with amplification efficiencies between 90 and 150% were used, and the Ct values in the liner range were used for calculation.

Promoter sequences with lengths no more than 2 kb of several genes involved in ABA and BR biosynthesis or signaling pathways, including *MdBIN2*, *MdBKI1*, *MdCDP*, *MdDWF4*, *MdMYC2*, *MdNCED3*, and *MdRD22*, were obtained from GDR. They were then used to detect the CACG motif, the core binding site of NAC transcription factor.

### 2.7. Growth Parameters

Morphological indexes were determined for transgenic and WT plants, 10 individual plants of each overexpression (OE) or WT line were randomly selected from their corresponding lines of 40 plants were used for the test. They included total height, stem diameter (15 cm above the base), root length, average length of 10 internodes (between the 20th and the 30th internodes from the apex of the main stem), and leaf area of the eighth leaf from the apex. We also calculated the percentage of plants (from all genotypes) that had lateral branches longer than 0.5 cm as well as their average length and number. Root diameters was examined according to the method described previously, using the WinRHIZO^®^ image analysis (V4.1 c; Regent Instruments, Quebec City, QC, Canada) [30].

### 2.8. Photosynthetic Characteristics

The net photosynthetic rate (Pn), transpiration rate (Tr), stomatal conductance (Gs), and intercellular CO_2_ concentration (Ci) were monitored from 9:00 to 11:00 a.m., using a portable Li-6400 system (LICOR, Huntington Beach, CA, USA). These measurements were conducted at 500 μmol photons m^−2^ s^−1^ and a constant airflow rate of 500 μmol s^−1^. The cuvette CO_2_ concentration was set at 500 μmol CO_2_ mol^−1^ air, with a vapor pressure deficit of 2.0–3.4 kPa. Data were recorded from eight fully expanded leaves at the same position on different plants of each line. Instantaneous water-use efficiency (WUEi) was calculated as the ratio between net photosynthesis and transpiration rate, i.e., WUEi = Pn / Tr [31].

### 2.9. Anatomical Structure Analysis

For histological analysis, leaf and young stem sections sampled from transgenic lines and WT plants were fixed in an FAA (formaldehyde-acetic acid-ethanol) solution (50% ethanol, 5% glacial acetic acid, and 3.7% formaldehyde) and placed under a vacuum for 30 min to remove air before standing for 24h. The samples were then dehydrated in absolute ethanol (10 min), rinsed twice in xylene (20 min each time), and embedded in paraffin. After sectioning with a microtome, the samples were stained with safranin and fast green according to the method described by Ma et al. [32]. The paraffin sections were then examined with a Nikon Eclipse Microscopy (Instech Co., Ltd., Kanagawa, Japan).

For stem analysis, lengths and widths were recorded for the epidermal cells, and the first layer of cortical parenchyma cells was measured in the longitudinal sections. From the transverse sections, the thicknesses of the cortex and diameter of the vascular cylinder were determined, as well as the density of cortical parenchyma cells and pith parenchyma cells. For leaf analysis, the thicknesses of the leaf, upper epidermis, palisade tissue, spongy tissue, and lower epidermis were measured from transverse sections. Each experiment was performed for four biological repeats.

### 2.10. Concentrations of Plant Hormones

The endogenous concentrations of IAA, GA, CTK, ABA, and BR were obtained using mature leaves from transgenic and WT lines harvested from the same position on each selected plant. The samples were immediately frozen in liquid nitrogen and stored at ‒80 °C. Extraction, purification, and determination of these hormones were performed with an indirect enzyme-linked immunosorbent assays (ELISAs), as described previously [33]. Briefly, 0.2 g leaf samples from each genotype were ground to powder in liquid nitrogen. They were extracted in 10 mL of an 80% (v/v) methanol extraction medium that contained 1 mM butylated hydroxytoluence. After drying under N_2_, the samples were dissolved. For quantifications, standard samples for each hormone were used to manufacture standard curves. The ELISAs were performed in a 96-well microtitration plates. Antibodies of each hormone were added to the corresponding wells. After incubation, the cells were washed and filled with the second antibodies before being incubated and washed again. The reaction process was started and then stopped, and color development was detected for each well, using an ELISA Reader at an optical density of A_490_. The concentration of each hormone was obtained by applying the method of Weiler et al. [34]. The antibodies used for ELISA were prepared and supplied by the Engineering Research Center of Plant Growth Regulator, China Agricultural University (Beijing, China). For each sample, we used five trees per genotype and performed three biological replicates.

### 2.11. Statistical Analysis

Most of the data are presented as means ± standard deviations. Significant differences (*p* < 0.05) among values were assessed by one-way ANOVA, followed by a Tukey’s multiple comparison test. Statistical computations were made with IBM SPSS Statistics 20 software (SPSS Inc., Chicago, IL, USA).

## 3. Results

### 3.1. Expression, Cloning, and Molecular Characterization of MdNAC1 Gene

The expression of the *MdNAC1* gene was tested in dwarfing and nondwarfing apple rootstocks. Expression of it was significantly higher in the three dwarfing rootstocks (M9, SH2, and T337) than that in the three nondwarfing rootstock (*Malus zumi*, *Malus pruifolia*, and *Malus robusta*) (Figure S1). These results suggested that it possible plays a role in apple dwarfing. This gene was selected for further study.

**Figure 1:**
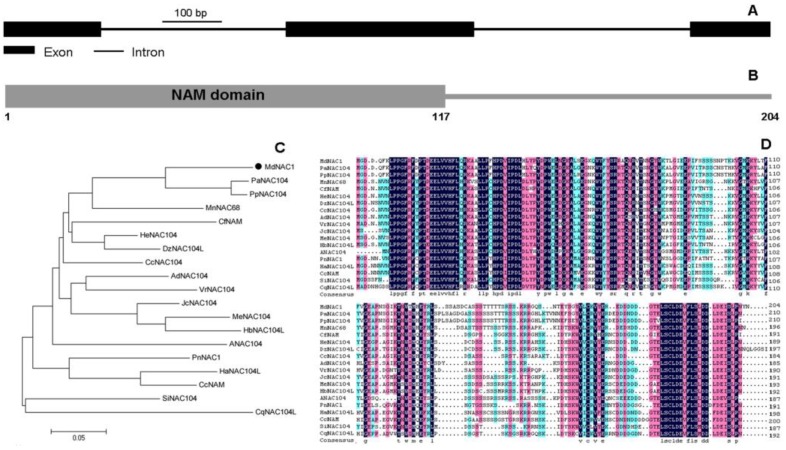
Gene structure and protein sequence analysis of *MdNAC1*. (A) Exons/introns of *MdNAC1* are displayed. (B) NAM domain detected in the N-terminus region of MdNAC1. (C) Phylogenetic analysis of MdNAC1 (marked by solid-black circle) and homologous proteins from other species. (D) Alignment of MdNAC1 and its homologous proteins. These different colors show the similarity degrees of the amino acids. Homologous protein sequences of MdNAC1 from 18 other species were used for phylogenetic analysis and alignment.

Using the online HMM program, we verified that a NAM domain (pfam02365), a conserved domain of NAC TFs, was detected in the N-terminus region of this protein (Figure 1B). This target gene was named *MdNAC1* and submitted to GenBank with Accession number MF401514.1. Its open reading frame (ORF) contains 615 nucleotides encoding a protein of 204 amino acids (Figure 1B). Structure analysis indicated that this gene has three exons and two introns (Figure 1A). Based on results from phylogenetic analysis and multiple alignments, MdNAC1 shares high similarity with homologues from other species (Figure 1C,D), including 79% similarity with PaNAC104 (XP_020421993.1) from *Prunus avium* and 77% similarity with PpNAC104 (XP_021826170.1) from *P. persica*. However, it shows only 59% similarity with ANAC104 from *Arabidopsis*.

### 3.2. MdNAC1 Is Mainly Located in the Nucleus and Has Transcriptional Activity

**Figure 2:**
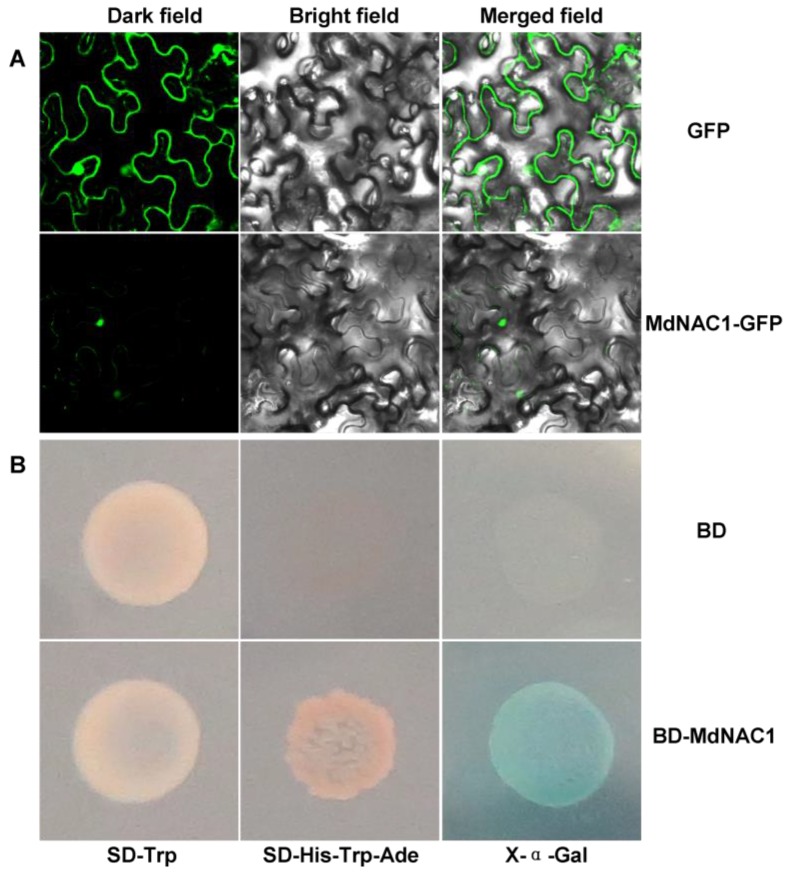
(A) Subcellular localization of MdNAC1. GFP (green fluorescent protein) or MdNAC1-GFP fusion protein were transiently expressed in *Nicotiana benthamiana* epidermal cells and analyzed by confocal microscopy. The green fluorescent signal in the dark field or merged field shows the localization of GFP or MdNAC1-GFP fusion protein. (B) Transcriptional activity analysis of MdNAC1 in yeast. The transformed yeast strain AH109 with both GAL4 DNA binding domain (BD) and BD-MdNAC1 fusion protein grows well on SD-Trp medium. The yeast with BD-MdNAC1 grows well, but not with BD, on SD-His-Trp-Ade and SD-His-Trp-Ade mediums with X-α-Gal. Growth on SD-Trp means that the protein is expressed in yeast stain AH109; growth on SD-His-Trp-Ade means that there is functional expression of His and Ade biosynthetic genes; and the blue colony is the indicator showing the expression of these genes. SD-Trp medium: synthetic, minimal lacking Trp; SD-His-Trp-Ade: SD medium lacking His, Trp, and Ade; X-α-Gal: SD-His-Trp-Ade medium plus X-α-Gal.

Free GFP protein was localized in the cytoplasm and nucleus of tobacco epidermis cells (Figure 2A). The MdNAC1‒GFP fusion protein was consistent with localization to the nucleus, with only subtle distribution in the plasma membrane (Figure 2A).

When transformed into yeast cells, yeast cells that harbored only BD protein (negative control) did not grow normally on SD-Trp-His mediums with or without X-α-Gal (Figure 2B). However, the BD‒MdNAC1 fusion protein activated growth of cells on those same types of media (Figure 2B), which suggested that MdNAC1 has transcriptional activity.

### 3.3. Dwarf Phenotype of the MdNAC1 Overexpression Apple Lines

To characterize the function of *MdNAC1*, we generated transgenic GL-3 apple plants that over-expressed *MdNAC1* and obtained four OE lines that were then verified by PCR (Figure S2A,B). Results from qRT-PCR analysis showed that expression of this gene was dramatically increased in all four OE lines when compared with the WT (Figure S2C). From this, we selected lines OE-1, OE-2, and OE-3 for further analysis.

**Figure 3:**
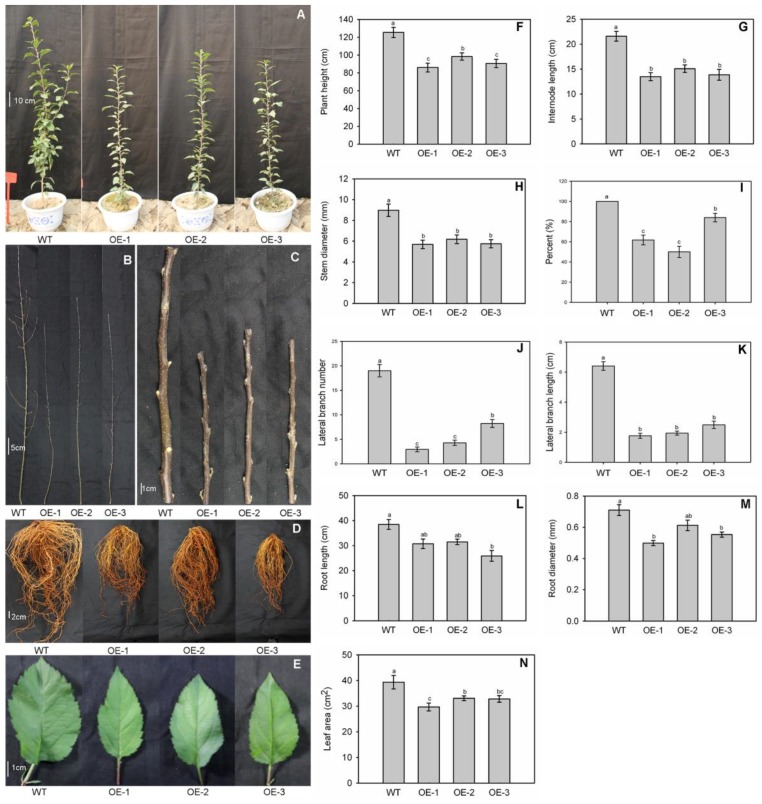
Dwarf phenotype in overexpression lines of *MdNAC1*. (**A**) Transgenic plants had smaller plant size and (**C**) internodes length. (**B**) They also featured changes in lateral branch architecture, smaller roots (**D**) and leaves (**E**), and (**F**) lower values for plant height, (**G**) internode length, (**H**) stem diameter, (**L**) root length, (**M**) root diameter, and (**N**) leaf area. (**I**) Transgenic plants had lower shoots with lateral branches, (**J**) lower lateral branches, and (**K**) shorter lateral branches. The data for Figure 3I–K are presented as means ± standard errors; The data for other Figures are presented as means ± standard deviations. Different letters above the bars indicate the significant difference among these data at a level of *p* < 0.05 according to Tukey’s multiple comparison test. Any data sharing a same letter means there is no significant difference at this significant level. a: Subset label showing one or more sets of data with the largest mean value(s); b: Subset label showing one or more sets of data with the second-largest mean value(s); c: Subset label showing one or more sets of data with the third-largest mean value(s).

After 90 d growth in the greenhouse, the transgenic plants were shorter than the WT (Figure 3A), i.e., 125 (WT) versus 86 (OE-1), 98 (OE-2), and 90 cm (OE-3) (Figure 3F). The OE plants also had much shorter internodes and thinner stems (Figure 3C). For comparison, those average internode lengths were 2.2 (WT), 1.4 (OE-1), 1.5 (OE-2), and 1.4 cm (OE-3) (Figure 3G). Average stem diameters were 5.7 (OE-1), 6.2 (OE-2), and 5.8 mm (OE-3), all of which were lower than that of the WT, i.e., 9.0 cm (Figure 3H).

The OE plants had smaller leaves (Figure 3E), with average leaf areas being 29.7 (OE-1), 33.1 (OE-2), and 32.8 (OE-3) versus 39.3 cm^2^ for the WT (Figure 3N). Branch architecture also differed among genotype. Overexpression of *MdNAC1* caused reduction in the number and length of lateral branches (Figure 3B). Only 50%–84% of the OE plants had lateral branches, whereas all of the WT plants did (100%) (Figure 3I). The average number of lateral branches per plant were 2.9 for OE-1, 4.3 for OE-2, and 8.2 for OE-3, while the number in WT was 19.0 (Figure 3J). Their average lengths were 1.8, 1.9, and 2.5 cm for OE-1, OE-2, and OE-3, respectively, versus 6.4 cm for the WT (Figure 3K).

The OE plants also had significantly shorter, smaller roots (Figure 3D). Whereas the WT average root length was 38.5 cm, those values were 30.7 cm for OE-1, 31.5 cm for OE-2, and 25.9 cm for OE-3 (Figure 3L). Finally, the average root diameters were 0.71 mm for the WT versus 0.50 cm, 0.61 cm, and 0.55 cm for OE-1, OE-2, and OE-3, respectively (Figure 3M).

### 3.4. Ultrastructure Analysis of Stems and Leaves

**Figure 4:**
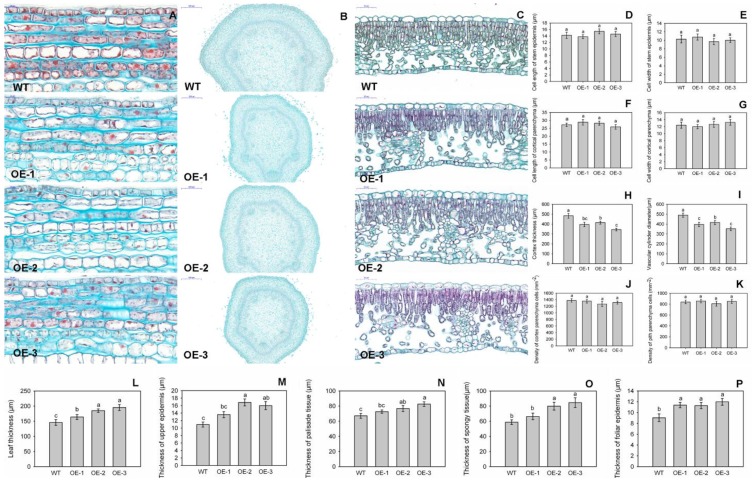
Anatomical structures of stems and leaves from transgenic and wild-type (WT) plants. Longitudinal paraffin sections (**A**) and transverse paraffin sections (**B**) of stems from transgenic and WT plants analyzed using a light microscopy. (**C**) Transverse paraffin sections of leaves from transgenic and WT plants. Genotypes did not differ significantly in the longitudinal lengths of stem epidermis cells (**D**), or stem cortical parenchyma cells (**E**), widths of epidermis cells (**F**), or stem cortical parenchyma cells (**G**), cell density of cortical parenchyma cells (**J**) or pith parenchyma cells (**K**). However, transgenic plants displayed smaller cortical thickness (**H**) and smaller vascular cylinder diameter (**I**), but had thicker in leaves (**L**), upper epidermis (**M**), palisade tissue (**N**), spongy tissue (**O**), and foliar epidermis (**P**) when compared with WT. Anatomical structures of stems or leaves were analyzed with four biological repeats. The data are presented as means ± standard deviations. Detailed information on statistics, please see Figure 3.

To determine whether this altered morphology for transgenic apple plants was associated with changes in cell size or tissue organization, we examined various tissue sections under a light microscope. Longitudinal sections of young stems revealed no significant differences in the sizes of epidermal cells and cortical parenchyma cells between the OE and WT samples (Figure 4A), based on their cell lengths and widths (Figure 4D–G). However, those stems were significantly thinner from the transgenics than from the WT (Figure 4B), with cortical thickness values of 396.1 μm for OE-1, 414.9 μm for OE-2, 344.4 μm for OE-3, and 483.6 μm for the WT (Figure 4H). The vascular cylinder diameters were also significantly lower in the OE lines, i.e., 673.3 μm for OE-1, 861.3 μm for OE-2, and 749.3 μm for OE-3, when compared with 955.3 μm in WT plants (Figure 4I). To determine whether cell numbers in the stems had been altered because of overexpression, we calculated cell densities for the parenchyma from the cortex and pith, but found no significant differences among genotypes (Figure 4J,K). Therefore, all of these data indicated that decreases in internode lengths and stem diameters were responsible for the decline in total cell numbers.

We were interested to learn that the leaves from OE lines were significantly thicker than those from WT plants (Figure 4C). Those measurements were 164.0 μm for OE-1, 184.8 μm for OE-2, 195.3 μm for OE-3, and 145.9 μm for the WT (Figure 4L). Meanwhile, values for the upper epidermis, palisade tissue, spongy tissue, and the lower epidermis were obviously higher for OE plants than for the WT (Figure 4M–P).

### 3.5. Photosynthesis and Gas Exchange Parameters

**Figure 5:**
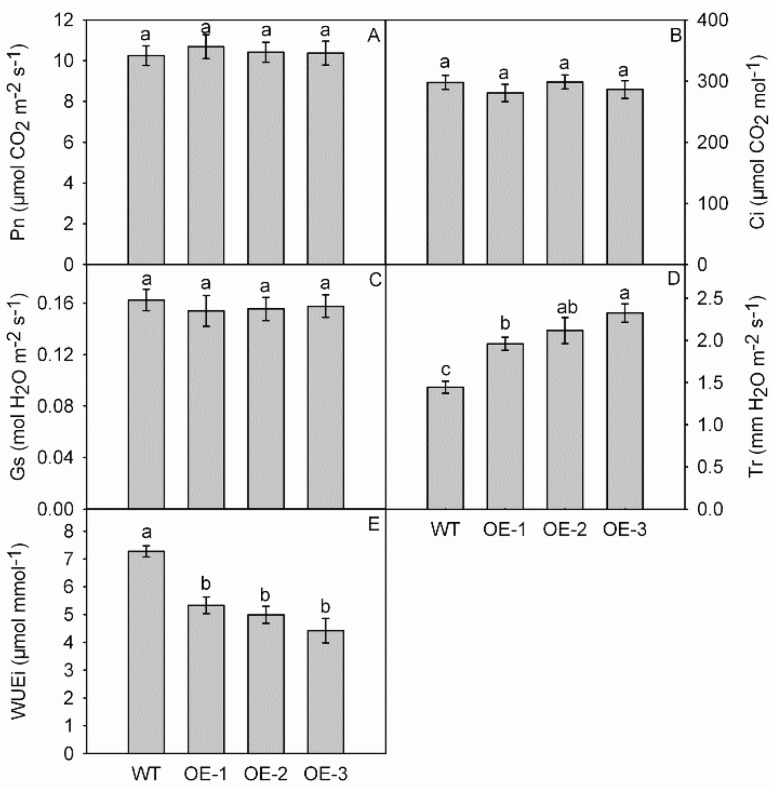
Photosynthetic parameters. Genotypes did not differ significantly in values for net photosynthetic rate (Pn) (**A**), intercellular CO_2_ concentration (Ci) (**B**), and stomatal conductance (Gs) (**C**). However, transgenic plants had higher transpiration rate (Tr) (**D**), but lower instantaneous water-use efficiency (WUEi) (**E**) when compared with wild-type (WT). Detailed information on statistics, please see Figure 3.

To investigate whether overexpression of *MdNAC1* would alter photosynthetic capacity, we compared the data for Pn, Ci, Gs, Tr, and WUEi between the WT and OE lines. Values did not differ significantly for Pn, Ci, or Gs (Figure 5A–C). However, Tr and WUEi measurements varied, with transpiration rates being 1.96 mmol H_2_O m^−2^s^−1^ in OE-1, 2.12 mmol H_2_O m^−2^s^−1^ in OE-2, and 2.32 mmol H_2_O m^−2^s^−1^ in OE-3, all of which were significantly higher than the 1.44 mmol H_2_O m^−2^s^−1^ determined for the WT (Figure 5D). Overexpression of *MdNAC1* led to significantly lower WUEi in the OE plants (5.33 μmol mmol^−1^, in OE-1; 4.99 μmol mmol^−1^, OE-2; 4.42 μmol mmol^−1^, OE-3) than in the WT (7.27 μmol mmol^−1^) (Figure 5E).

### 3.6. Overexpression of MdNAC1 Reduces the Biosynthesis of ABA and BR

**Figure 6:**
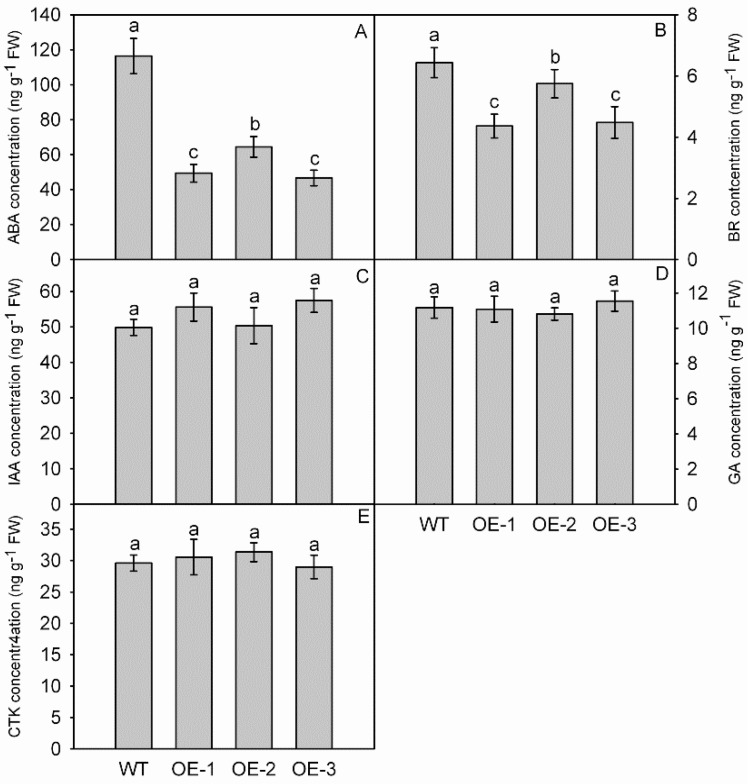
Concentrations of abscisic acid (ABA) (**A**), brassinosteroid (BR) (**B**), indole acetic acid (IAA) (**C**), gibberellin (GA) (**D**), and cytokinins (CTK) (**E**) in transgenic and wild-type (WT) plants. The data are presented as means ± standard deviations. Detailed information on statistics, please see Figure 3.

While the concentrations of IAA, GA, and CTK differed only slightly between OE and WT plants (Figure 6C–E), the levels of ABA and BR were significantly lower in the OE lines (Figure 6A,B). For ABA, those values were 49 ng g^−1^ FW (OE-1), 64 ng g^−1^ FW (OE-2), and 47 ng g^−1^ FW (OE-3) versus 116 ng g^−1^ FW in the WT (Figure 6A). Concentrations of BR ranged from 4.4 ng g^−1^ FW to 5.7 ng g^−1^ FW in the OE plants and were significantly lower than the level obtained for the WT, i.e., 6.43 ng g^−1^ FW (Figure 6B).

**Figure 7:**
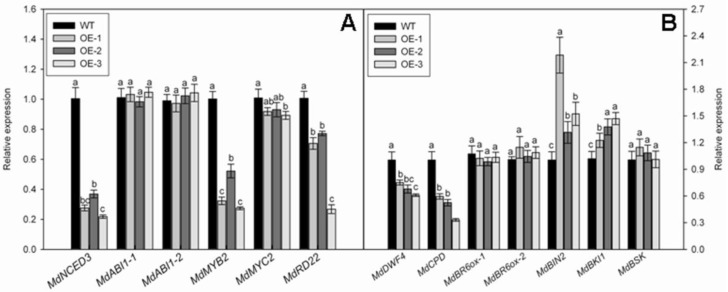
(**A**) Relative expressions of genes related to biosynthesis and (**B**) signal pathways for ABA and BR by quantitative real-time PCR (qRT-PCR) analysis. The data are presented as means ± standard deviations. Detailed information on statistics, please see Figure 3.

We performed qRT-PCR analyses to examine the expression of genes involved in ABA production and its signal transduction. They included *MdNCED3*, *MdABI1-1*, *MdABI1-2*, *MdMYB2*, and *MdMYC2*. Their homologous genes in *Arabidopsis* encode the limiting enzyme in ABA biosynthesis (*NCED3*) [35]; negative regulators (*ABI1-1* and *ABI1-2*) [36]; positive regulators (*MYB2* and *MYC2*) [37]; and an ABA-response marker (*RD22*) [38]. Transcript levels of *MdNCED3, MdMYB2*, and *MdRD22* were significantly lower in OE lines than in the WT, and that of *MdMYC2* was slightly lower in OE lines than in the WT, while the expression of *MdABI1-1* and *MdABI1-2* was not obviously different among genotypes (Figure 7A). 

Several genes were related in BR biosynthesis or signal pathways, including genes for three rate-limiting enzymes, i.e., *DWF4*, *CPD*, and *BR6ox* [6,39,40]; two negative regulators, *BIN2* and *BRI1* [6]; and a BR signaling kinase gene, *BSK* [6]. We monitored the expression levels of their corresponding homologous genes in our over-expressed and WT apple plants. Our qRT-PCR results revealed no significantly genotypic differences in transcript levels for *MdBR6ox-1*, *MdBR6ox-2*, or *MdBSK*. However, expression of *MdDWF4* and *MdCDP* was significantly lower in OE lines than in the WT, while expression of *MdBKI1* and *MdBIN2* increased in those OE plants (Figure 7B). The liner range and amplification efficiency of all the primers used in qRT-PCR analysis are shown in Table S5. The calibration curves of these primers are presented in Figure S4.

## 4. Discussion

The plant NAC TFs play important roles in diverse processes, such as shoot formation [41], lateral roots development [42], and the synthesis of xylem lignocellulose [17] and secondary cell walls [43]. Overexpression of a NAC TF gene, *ANAC036*, confers a dwarf phenotype in *Arabidopsis* [15]. In poplar, overexpression of *PopNAC154* results in reduced height growth [21]. To understand how *MdNAC1* might function in apple, we generated transgenic overexpressing plants and determined that they were significantly shorter than the WT because their internodes were not as long. In addition, the transgenics had shorter and narrower roots. This reduced root size, as part of the dwarf phenotype, might have caused the root system to have weaker assimilation capacity [7], perhaps explaining, in part, the dwarf phenotype observed for transgenic apple plants that over-expressed *MdNAC1*. However, although the transgenics were shorter in root and shoot, they showed a dwarfing character that may be useful in dwarf fruit production.

When we examined the photosynthetic parameters of our test plants, we noted that the rate of net photosynthesis, as well as values for intercellular CO_2_ concentrations and stomatal conductance, did not differ significantly among genotypes. However, the transpiration rate was significantly higher in OE plants than in the WT. This increase in transpiration resulted in less water-use efficiency. In addition, because the transgenic plants had fewer and shorter lateral branches, they then produced fewer leaves and had smaller total leaf areas when compared with samples from the WT. Therefore, although the net photosynthesis rate was not changed in the OE lines, the fact that they had fewer and smaller leaves meant that whole-plant capacity for assimilation was diminished, thereby leading to an overall reduced plant size.

Reductions in cell division and elongation can lead to a dwarf phenotype [15,44]. When the *Arabidopsis* gene *gai* is over-expressed in apple, plants are smaller than normal and there is an obvious decrease in the lengths of stems and internodes [4]. Our transgenic apples were shorter than the WT, and they had shorter internodes and roots. Many dwarf phenotypes are an outcome of changes in both cell size and cell numbers. For example, colchicine-induced autotetraploid apple plants exhibit dwarfing, and they have shorter cortical parenchyma cells in the vertical orientation [6]. In contrast, the longitudinal stem cells are the same length in the dwarf *Arabidopsis axr1* mutant (*axr1-12*) and corresponding WT plants (Lincoln et al. 1990) [45]. Therefore, it can be difficult to determine the reasons for these different dwarf phenotypes. We found here that overexpression of *MdNAC1* did not change the size of cells in the stem, but it did lead to significant decreases in the thickness of the cortex and the diameter of the vascular cylinder. Therefore, we conclude that these transgenic apple plants were smaller than the WT, mainly because of a reduction in cell numbers rather than cell size.

Phytohormones IAA, GA, CTK, ABA, and BR can be regulatory factors for dwarfism because changes in their concentrations, transport, or signaling can elicit that phenotype [3,8,46,47]. However, we detected no obvious differences in the amounts of IAA, GA, or CTK between the OE and WT plants. Only the endogenous levels of ABA and BR were significantly decreased in OE plants. This indicated that *MdNAC1* alters the accumulations of ABA and BR, thereby suppressing plant growth.

Although ABA functions as a growth inhibitor [10], our investigation showed that concentrations of that hormone were significantly lower in the dwarfed transgenic plants than in the WT. This suggested that a certain level of ABA is necessary to support plant growth. In fact, endogenous ABA can help maintain rather than inhibit shoot and root development [48]. For example, mutation of *ABA1* in *Arabidopsis* indicates that ABA acts as a growth promoter during the vegetative stage [49]. Furthermore, shoot growth is substantially inhibited in the ABA-deficient *Arabidopsis* mutant *aba2-1* [50]. In tomato, the ABA-deficient *notabilis/flacca* double mutant is associated with smaller plants and fruits, implying that ABA stimulates cell enlargement during the stages of fruit formation [51].

Brassinosteroid also has an important connection with plant dwarfism. Overexpression of *MdWRKY9* confers a dwarf phenotype in M26 apple rootstocks due to inhibited BR synthesis. That gene represses the expression of *MdDWF4*, which encodes a BR rate-limiting synthetase and leads to a lower concentration of BR in transgenic M26 plants [7]. We also detected lower levels of BR in our OE plants. Internode lengths are related to BR concentrations. For example, the rice BR-deficient mutant *d2* has shorter internodes compared with WT plants [52]. Decreased synthesis of BR leads to a dwarf phenotype in colchicine-induced autotetraploid apple plants, and cortical parenchyma cells are shorter, vertically, in autotetraploids than in diploids [6]. In *Zea mays*, the loss-of-function mutation of *na1*, a *DET2* homolog in the BR biosynthesis pathway, is also linked to shorter internodes [53]. Therefore, overexpression of *MdNAC1* conferred shorter internode lengths in our transgenic apple, probably because they had lower BR concentrations.

We used qRT-PCR analysis to investigate whether overexpression of *MdNAC1* reduces the expression of genes involved in the biosynthesis and signal transduction pathways for ABA and BR. Transcript levels for *MdNCED3*, *MdDWF4*, and *MdCDP* were significantly lower in OE plants, which was consistent with the decreased amounts of endogenous ABA and BR measured in those samples. Several genes in their signal transduction pathways were also suppressed in OE plants. These results demonstrated that overexpression of *MdNAC1* in apple modifies the ABA and BR pathways.

Several NAC TFs are involved in plant dwarfism and/or growth inhibition in plants. Those responses can also be prompted by overexpression of *Arabidopsis* NAC genes, including *XND1*, *VND7*, and *ANAC036* [15,17,18]. *OsNAC6* and *OsSWN1* can also confer dwarf and semi-dwarf phenotypes, respectively, in rice [19,20]. In poplar, a woody plant, *PopNAC122* inhibits height growth and the size and number of cells [29]. The NAC genes are also active in the ABA and BR pathways. In *Arabidopsis*, *ATAF1* attenuates ABA signaling to mediate efficient resistance to penetration by pathogens [54]. Expression of *AtNAP* probably elevates the level of ABA during leaf senescence [55]. In rice, *OsNAP* functions in the abiotic stress response by regulating ABA-mediated pathways [56], while JA2 increases the production of that hormone by activating the expression of *NCED1* in transgenic tomato plants [57]. The *Arabidopsis* NAC TF JUB1 inhibits BR synthesis by directly reducing the expression of *DWF4* and its orthologs in *Arabidopsis* and tomato, thereby leading to dwarf phenotypes in both of those species [58,59]. We also noted here that the concentrations of ABA and BR were diminished in the transgenic apple plants, as were the levels of expression for genes involved in the biosynthesis of ABA (*MdNCED3*) and BR (*MdDWF4* and *MdCDP*), as well as genes involved in the signaling pathways for ABA (*MdMYB2*, *MdMYC2*, and *MdRD22*) and BR (*MdBIN2* and *MdBKI1*). Therefore, we conclude that *MdNAC1* regulates the expressions of these apple genes to reduce the levels of ABA and BR, ultimately leading to the dwarf phenotype.

The NAC TFs preferentially bind to the NAC core binding site of CACG in the promoters of their target genes [60]. At least one CACG motif has been identified in the promoter regions of *MdBIN2*, *MdBKI1*, *MdCDP*, *MdDWF4*, and *MdMYC2* (Figure S3). Six CACG motifs are also distributed in the promoter regions of *MdNCED3* and *MdRD22* (Figure S3). This implies that MdNAC1 may directly bind to the promoters of those genes to regulate the ABA and BR pathways. However, further research is needed to obtain direct evidence that MdNAC1 functions upstream in those pathways.

## 5. Conclusions

We determined that overexpression of *MdNAC1* confers a dwarf phenotype in transgenic apple plants. This was manifested by a reduction in the overall size of the plant, including its height, internode and root lengths, total leaf area, and the diameters of stems and roots. Furthermore, we noted that the levels of endogenous ABA and BR were decreased in our transgenics, along with the expression of genes involved in their pathways. All of these findings suggest that MdNAC1 confers a dwarf phenotype by regulating the ABA and BR pathways in growing plants growth. Thus, *MdNAC1* can be a useful molecular tool for improving the dwarf apple plants in modern apple production.

## Supplementary Materials

The following are available online at http://www.mdpi.com/2073-4425/9/5/229/s1. Figure S1: Relative expressions of *MdNAC1* in three non-dwarfing rootstocks and three dwarfing rootstocks. Different letters above the bars indicate the significant difference among these data at a level of p < 0.05 according to Tukey’s multiple comparison test. Any data sharing a same letter means there is no significant difference at this significant level. a: Subset label showing one or more sets of data with the largest mean value(s); b: Subset label showing one or more sets of data with the second-largest mean value(s); c: Subset label showing one or more sets of data with the third-largest mean value(s). Figure S2: (A) PCR detection of *MdNAC1* in overexpression lines using vector-specific primers of *att*B1 and *att*B2; (B) PCR detection of *MdNAC1* in overexpression lines using one gene-specific primer of *MdNAC1*F and one vector-specific primer of *att*B2 and (C) relative expression analysis of *MdNAC1* by qRT-PCR. Different letters above the bars indicate the significant difference among these data at a level of p < 0.05 according to Tukey’s multiple comparison test. Any data sharing a same letter means there is no significant difference at this significant level. a: Subset label showing one or more sets of data with the largest mean value(s); b: Subset label showing one or more sets of data with the second-largest mean value(s); c: Subset label showing one or more sets of data with the third-largest mean value(s); d:Subset label showing one or more sets of data with the forth-largest mean value(s) Figure S3: Schematic diagram of promoters of seven genes involved in ABA and BR pathways. Black arrows show the presence of NAC core binding site (CACG). The translational start site (ATG) is shown at position +1. Figure S4: The calibration curves of all the primers used in the qRT-PCR analysis. Table S1: Primers used for gene clone, vector construction, and PCR confirmation. Table S2: NAC proteins of 19 species used for multiple alignments and construction of phylogenetic tree. Table S3: Primers used for qRT-PCR. Table S4: Identification of *Arabidopsis* homologous genes in apple (*Malus domestica*). Table S5: Liner range and amplification efficiency of primes used in qRT-PCR.
