# Supplementary figures and images for: Overexpression of a Novel Apple NAC Transcription Factor Gene, MdNAC1, Confers the Dwarf Phenotype in Transgenic Apple (Malus domestica)
Source: Genes (Basel). 2018 Apr 27;9(5):229. doi: 10.3390/genes9050229 (PMC5977169; doi:10.3390/genes9050229)

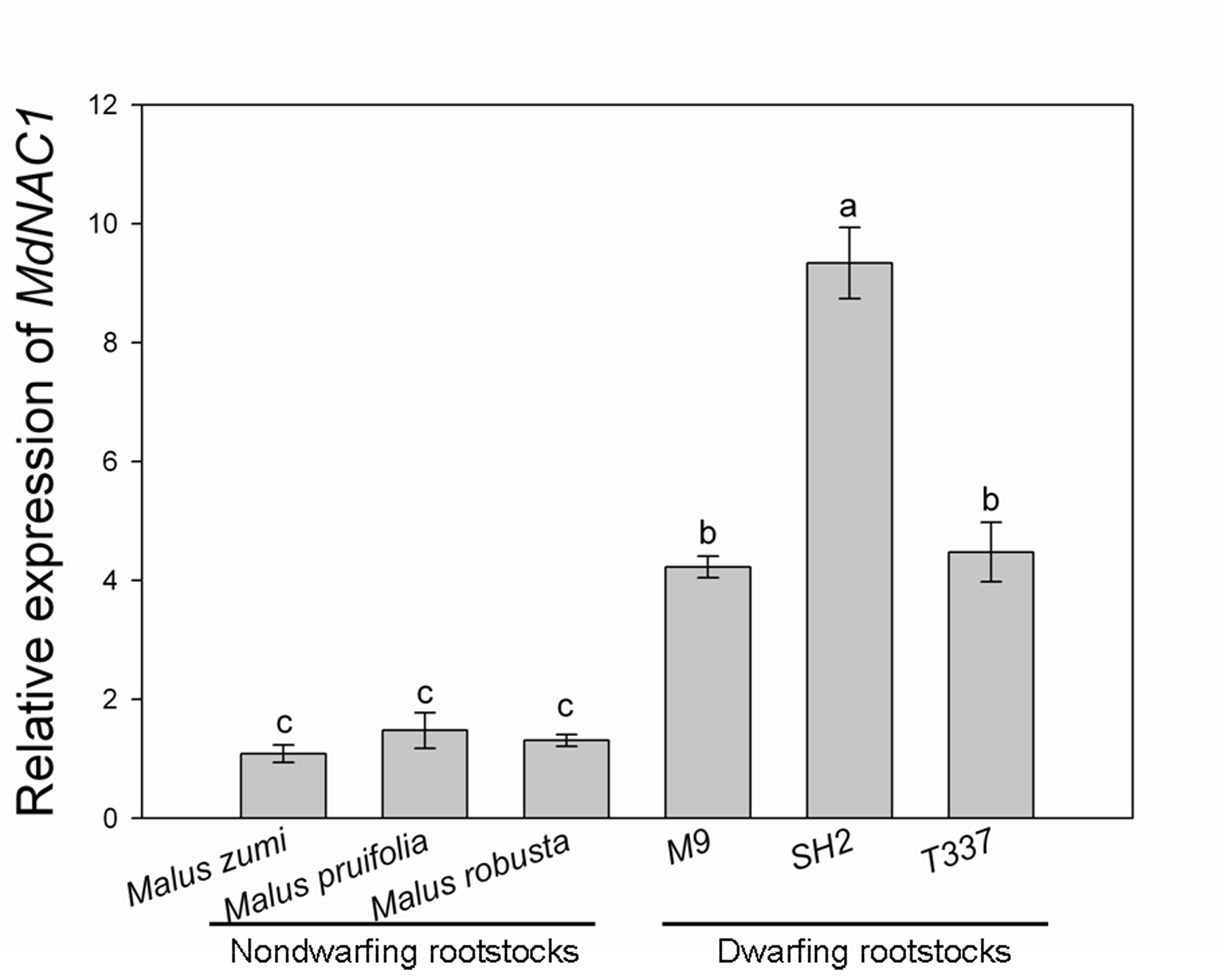

Supplement: Supplementary file 1 [file genes-09-00229-s001.zip › Supplementary Files/Figure S1.tif]

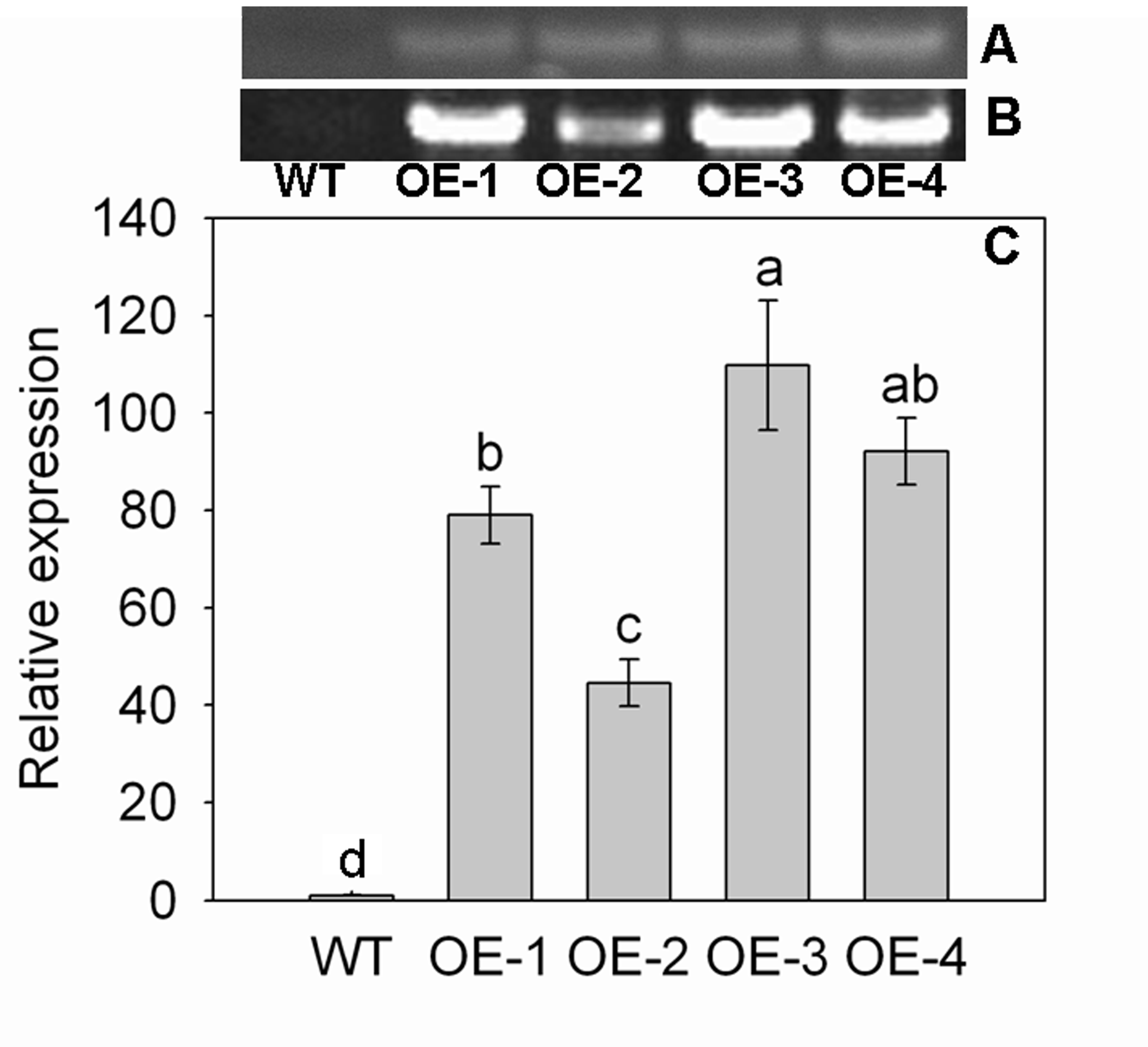

Supplement: Supplementary file 1 [file genes-09-00229-s001.zip › Supplementary Files/Figure S2.tif]

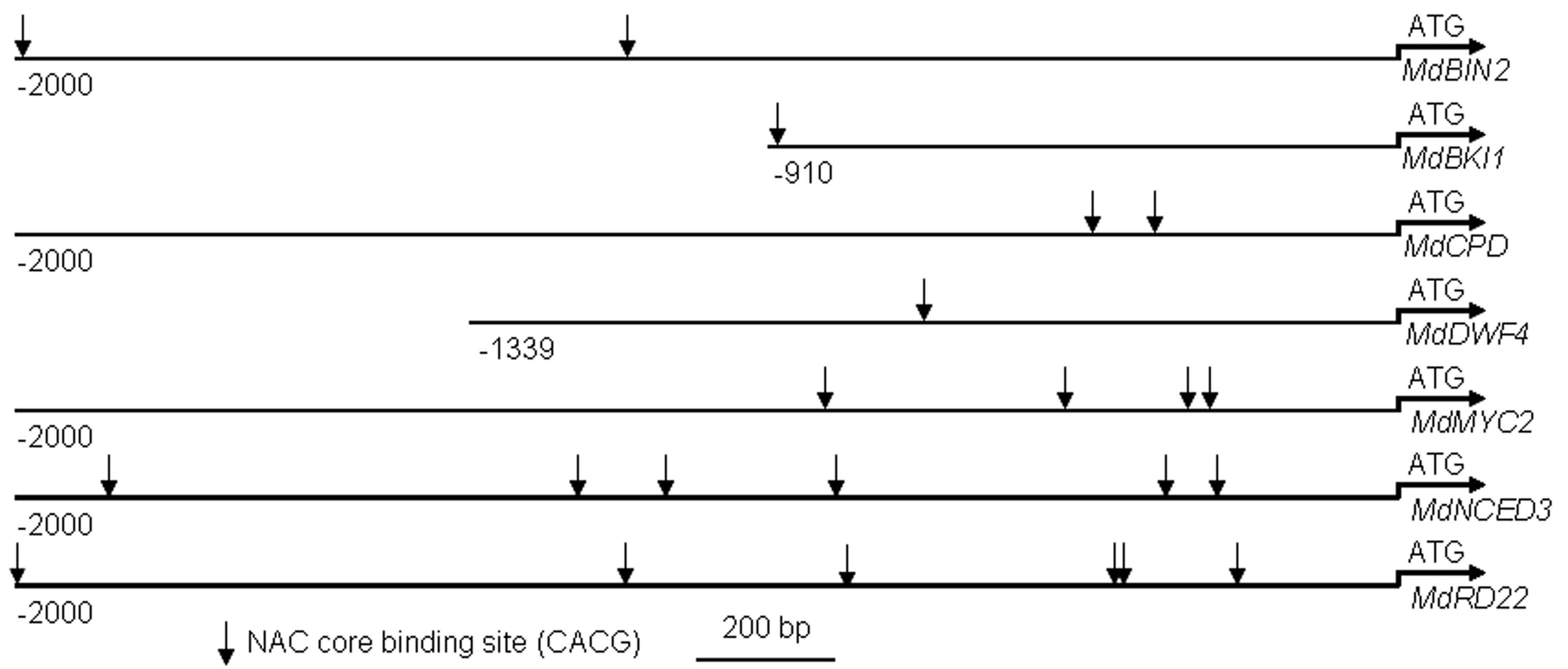

Supplement: Supplementary file 1 [file genes-09-00229-s001.zip › Supplementary Files/Figure S3.tif]

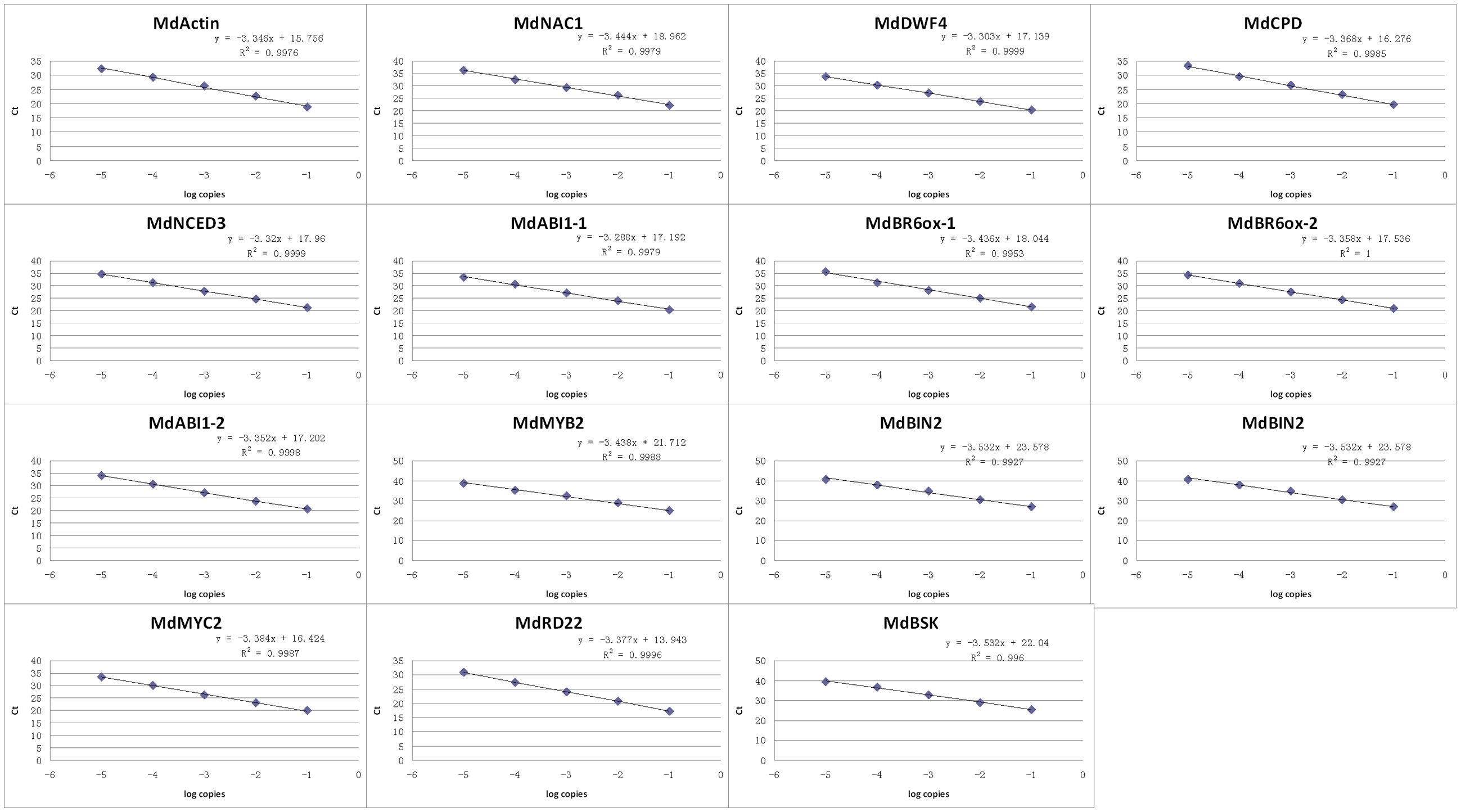

Supplement: Supplementary file 1 [file genes-09-00229-s001.zip › Supplementary Files/Figure S4.tif]
