# Supplementary material for: Overexpression of a Novel Apple NAC Transcription Factor Gene, MdNAC1, Confers the Dwarf Phenotype in Transgenic Apple (Malus domestica)
Source: Genes (Basel). 2018 Apr 27;9(5):229. doi: 10.3390/genes9050229 (PMC5977169; doi:10.3390/genes9050229)
Supplement: Supplementary file 1 [file genes-09-00229-s001.zip › Supplementary Files/Table S1.doc]

Table S1. Primers used for gene clone, vector construction and PCR confirmation.

| Primer name | Primer (5'-3') |
| --- | --- |
| *MdNAC1*cF | TCTTGTTATTGTCTGATATTCGTTCC |
| *MdNAC1*cR | TCTGTCCTTTAATTTTCCCCTTG |
| *MdNAC1*F | ACAAGTTTGTACAAAAAAGCAGGCTATGGGAGACGACCAGTTTA |
| *MdNAC1*R | ACCACTTTGTACAAGAAAGCTGGGTTCATTATAATTTGGCAAACTTA |
| *MdNAC1*slF | ACAAGTTTGTACAAAAAAGCAGGCTATGGGAGACGACCAGTTTA |
| *MdNAC1*slR | ACCACTTTGTACAAGAAAGCTGGGTTCATTATAATTTGGCAAACTTA |
| *MdNAC1*tfF | GGAATTCATGGGAGACGACCAGTTTAAGCT |
| *MdNAC1*tfR | CGGGATCCCTAATTATAATTTGGCAAACTT |
| *att*B1 | ACAAGTTTGTACAAAAAAGCAGGCT |
| *att*B2 | ACCACTTTGTACAAGAAAGCTGGGT |
| *MdNAC*F | ATGGGAGACGACCAGTTTAAGCTC |
