# Supplementary material for: Overexpression of a Novel Apple NAC Transcription Factor Gene, MdNAC1, Confers the Dwarf Phenotype in Transgenic Apple (Malus domestica)
Source: Genes (Basel). 2018 Apr 27;9(5):229. doi: 10.3390/genes9050229 (PMC5977169; doi:10.3390/genes9050229)
Supplement: Supplementary file 1 [file genes-09-00229-s001.zip › Supplementary Files/Table S2.doc]

Table S2. NAC proteins of 19 different species used for multiple alignments and construction of phylogenetic tree.

| NAC protein | Accession number | Species |
| --- | --- | --- |
| AdNAC104 | XP_015943391.1 | *Arachis duranensis* |
| ANAC104 | NP_201258.2 | *Arabidopsis thaliana* |
| CcNAC104 | XP_021896566.1 | *Carica papaya* |
| CcNAM | KVH93447.1 | *Cynara cardunculus* |
| CfNAM | GAV81136.1 | *Cephalotus follicularis* |
| CqNAC104L | XP_021751277.1 | *Chenopodium quinoa* |
| DzNAC104L | XP_022749101.1 | *Durio zibethinus* |
| HaNAC104L | XP_021988499.1 | *Helianthus annuus* |
| HbNAC104L | XP_021643780.1 | *Hevea brasiliensis* |
| HeNAC104 | XP_021287608.1 | *Herrania umbratica* |
| JcNAC104 | XP_012090474.1 | *Jatropha curcas* |
| MdNAC1 | MF401514.1 | *Malus domestic* |
| MeNAC104 | XP_021627814.1 | *Manihot esculenta* |
| MnNAC68 | XP_010103879.1 | *Morus notabilis* |
| PaNAC104 | XP_021826170.1 | *Prunus avium* |
| PnNAC1 | ASR92151.1 | *Panax notoginseng* |
| PpNAC104 | XP_020421993.1 | *Prunus persica* |
| SiNAC104 | XP_011092697.1 | *Sesamum indicum* |
| VrNAC104 | XP_014492319.1 | *Vigna radiata* |
