# Supplementary material for: Overexpression of a Novel Apple NAC Transcription Factor Gene, MdNAC1, Confers the Dwarf Phenotype in Transgenic Apple (Malus domestica)
Source: Genes (Basel). 2018 Apr 27;9(5):229. doi: 10.3390/genes9050229 (PMC5977169; doi:10.3390/genes9050229)
Supplement: Supplementary file 1 [file genes-09-00229-s001.zip › Supplementary Files/Table S3.doc]

Table S3. Primers used for qRT-PCR.

| Gene name | Accession number in GDR | Forward primer (5'-3') | Reverse primer (5'-3') |
| --- | --- | --- | --- |
| *MdNAC1* | MDP0000240094 | CTTCGTCAGCCTCAGATTGT | CCACTGTATTCTGTATTGGATTG |
| *MdNCED3* | MDP0000228070 | CACGACGAGAAGGAGTGGAAG | CGAGGGCAGCTGAATTGTAG |
| *MdABI1-1* | MDP0000893203 | GGATTTCATCACAAACCAGGAGG | CCGTTCCGTTGACATGGTATC |
| *MdABI1-2* | MDP0000647467 | AAGTGTGTGACCTTGCTCGG | GGGATCAATTCCTTCGCCTC |
| *MdMYB2* | MDP0000812154 | GCCTCATCGGACTCGTTTGG | GCAGCAGTAGTAGGGGTGTAATAATC |
| *MdMYC2* | MDP0000029168 | CAACAAGCCACCGTGAAAGC | CATCGCCAACTTTGGAATGTAGA |
| *MdRD22* | MDP0000228673 | CGTAAACACAGGGCACAAGG | GTTGGACGAAAATGGGATGGAT |
| *MdDWF4* | MDP0000498540 | GAAGGATGTTCGGTACAAAGGG | GGATTGAAGTGTTGCGGGTG |
| *MdCPD* | MDP0000313816 | AGAGGAGGGAGGAGAGCGAAG | CAAAGCAAGCAAGAAATCCACA |
| *MdBR6ox-1* | MDP0000286141 | GATTCTGGGTCCGAGGTCG | AGGTTCTTGATGTGTTGCTCCA |
| *MdBR6ox-2* | MDP0000307964 | CAACATGGATTGTGGGTAGCC | AGAGGAGGGAGGAGAGCGAAG |
| *MdBIN2* | MDP0000295137 | ACATTCCGAGTTCTTTGGGGT | CGATTTGTGCTGAGTCTGGTG |
| *MdBKI1* | MDP0000124873 | GGACATCTCCGACGAATAGTGG | TGGGCTTCTCTTCTGTGGCA |
| *MdBSK* | MDP0000092692 | CTTGCTTCCAAATGTCTTCAGTCT | TTTTCGGCACCTTCCTCATC |
| *MdActin* | EB136338.1^a^ | GGCTGGATTTGCTGGTGATG | TGCTCACTATGCCGTGCTCA |

^a^ Accession number of *MdActin* in GenBank.
