# Supplementary material for: Overexpression of a Novel Apple NAC Transcription Factor Gene, MdNAC1, Confers the Dwarf Phenotype in Transgenic Apple (Malus domestica)
Source: Genes (Basel). 2018 Apr 27;9(5):229. doi: 10.3390/genes9050229 (PMC5977169; doi:10.3390/genes9050229)
Supplement: Supplementary file 1 [file genes-09-00229-s001.zip › Supplementary Files/Table S4.doc]

Table S4. Identification of *Arabidopsis* homologous genes in apple (*Malus domestica*).

| Genes in *Arabidopsis thaliana* | Homologous genes in apple | E-Value | Protein similarity (%) |
| --- | --- | --- | --- |
| *NCED3* | *MdNCED3* | 0 | 67.31 |
| *ABI1* | *MdABI1-1* | 8.28E-123 | 49.25 |
| *ABI1* | *MdABI1-2* | 1.96E-74 | 36.52 |
| *MYB2* | *MdMYB2* | 6.65E-55 | 34.35 |
| *MYC2* | *MdMYC2* | 0 | 54.11 |
| *RD22* | *MdRD22* | 3.32E-118 | 55.98 |
