# Supplementary material for: Overexpression of a Novel Apple NAC Transcription Factor Gene, MdNAC1, Confers the Dwarf Phenotype in Transgenic Apple (Malus domestica)
Source: Genes (Basel). 2018 Apr 27;9(5):229. doi: 10.3390/genes9050229 (PMC5977169; doi:10.3390/genes9050229)
Supplement: Supplementary file 1 [file genes-09-00229-s001.zip › Supplementary Files/Table S5.doc]

Table S5. Liner range and amplification efficiency of primes used in qRT-PCR.

| Gene | Liner range (minimum to maximum) | Amplification efficiency (%) |
| --- | --- | --- |
| *MdActin* | 18.81-32.22 | 99.0 |
| *MdNAC1* | 22.16-36.23 | 95.1 |
| *MdNCED3* | 21.23-34.59 | 100.1 |
| *MdABI1-1* | 20.24-33.36 | 101.4 |
| *MdABI1-2* | 20.62-33.99 | 98.8 |
| *MdMYB2* | 24.95-38.86 | 95.4 |
| *MdMYC2* | 19.98-33.46 | 97.5 |
| *MdRD22* | 17.36-30.94 | 97.8 |
| *MdDWF4* | 20.42-33.69 | 100.8 |
| *MdCPD* | 19.56-33.28 | 98.1 |
| *MdBR6ox-1* | 21.64-35.69 | 95.5 |
| *MdBR6ox-2* | 20.88-34.28 | 98.5 |
| *MdBIN2* | 26.83-40.11 | 91.9 |
| *MdBKI1* | 19.86-33.67 | 96.3 |
| *MdBSK* | 25.34-39.16 | 92.2 |
